# Effects of pulmonary rehabilitation training based on WeChat App on pulmonary function, adverse mood and quality of life of COVID-19 patients: A protocol for systematic review and meta-analysis

**DOI:** 10.1097/MD.0000000000026813

**Published:** 2021-08-06

**Authors:** Lili Ding, Zhiyu Xu, Zhongyan Zhao, Haiping Li, Aihong Xu

**Affiliations:** a Department of Respiratory and Critical, Hainan General Hospital Hainan Affiliated Hospital of Hainan Medical University, Haikou, Hainan Province, China; b Department of Critical Care, Hainan General Hospital Hainan Affiliated Hospital of Hainan Medical University, Haikou, Hainan Province, China; c Department of Neurology, Hainan General Hospital Hainan Affiliated Hospital of Hainan Medical University, Haikou, Hainan Province, China; d Department of Geriatrics, Sanya People's Hospital, Hainan Province, Sanya, Hainan Province, China.

**Keywords:** coronavirus disease 2019, meta-analysis, protocol, pulmonary rehabilitation training, WeChat

## Abstract

**Background:**

Coronavirus disease 2019 (COVID-19) as a fatal epidemic has swept across the world, especially in India where the epidemic situation is the most serious. For COVID-19 patients, pulmonary rehabilitation training plays a significant role. However, it is still a controversial issue regarding the efficacy of WeChat APP-based pulmonary rehabilitation training in improving lung function, quality of life and bad mood of COVID-19 patients. To clarify this issue, a meta-analysis was conducted in this present study, so as to provide a basis for rehabilitation guidance of COVID-19 patients.

**Methods:**

We systematically searched PubMed, medRxiv, Web of Science, Scopus, Chinese Science Citation Database, China National Knowledge Infrastructure, Chinese Biomedical Literature Database, Chinese Scientific Journal Database, and Wan-fang databases in May 2021 to identify randomized controlled trials and evaluate the effects of WeChat APP-based pulmonary rehabilitation training for COVID-19. Two researchers independently carried out data extraction. On the other hand, literature quality evaluation on the quality and meta-analysis of the included literature was performed with Revman5.3 software.

**Results:**

The results of this meta-analysis will be submitted to a peer-reviewed journal for publication.

**Conclusion:**

This study will provide reliable evidence-based evidence on the effects of WeChat APP-based pulmonary rehabilitation training on lung function, bad mood, and quality of life in patients with COVID-19.

**Ethics and dissemination:**

Ethical approval was not required for this study. The systematic review will be published in a peer-reviewed journal, presented at conferences, and shared on social media platforms.

**OSF Registration number:**

DOI 10.17605/OSF.IO/MKXCH.

## 1 Introduction

Pulmonary rehabilitation training for patients with coronavirus disease 2019 (COVID-19) can improve the symptoms of dyspnea, optimize the oxygen transport capacity of patients’ lungs, effectively remove lung secretions, increase patients’ exercise endurance, improve the ability of daily living, and shorten the length of hospital stay.^[1]^ The pulmonary rehabilitation program for patients with COVID-19 involves physical and psychological aspects society and other aspects, and the implementation of the program is a long-term process.

Pulmonary rehabilitation training is a comprehensive rehabilitation with exercise as the core, which is mainly aimed at patients with lung diseases.^[2,3]^ Pulmonary rehabilitation training improves the physical and psychological status of patients with chronic respiratory diseases through exercise training, education and behavioural change, and encourages long-term adherence to health-promoting behaviours.^[4]^ Generally, the home lung rehabilitation training lasts for 8 to 12 weeks.^[5]^ However, to ensure the effectiveness of pulmonary rehabilitation, patients need long-term maintenance training. Pulmonary rehabilitation training has many limitations, including insufficient resources for pulmonary rehabilitation, low proportion of medical insurance allocation, and insufficient professional health care providers.^[6]^ Furthermore, other factors such as transportation, population movement, distance, and training location also prevent patients from requesting, participating in, and adhering to pulmonary rehabilitation training.^[7]^

Given that patients with COVID-19 need to be treated in isolation or isolated at home, it is particularly important to take appropriate guidance methods to train patients in lung rehabilitation. With the popularity of electronic products, mobile health management has been gradually applied. WeChat is a mobile Internet technology communication application launched by Tencent, and it can send text, voice, video and pictures over the network.^[8–10]^ In recent years, WeChat has been applied in the continuous care of patients after discharge, thus strengthening doctor-patient communication and improving the compliance of treatment after discharge.^[11–14]^

There is not enough evidence to support that WeChat APP-based pulmonary rehabilitation training can improve lung function, adverse mood and quality of life in COVID-19 patients. Therefore, this study aimed to evaluate the effects of WeChat APP-based lung rehabilitation training on lung function, adverse mood and quality of life in COVID-19 patients, and to provide reference for non-drug intervention in COVID-19 patients.

## 2 Methods

### 2.1 Study registration

The systematic review protocol has been registered in open science framework, and the registration number is DOI 10.17605/OSF.IO/MKXCH. The consent of this protocol report is based on the Preferred Reporting Items for Systematic Reviews and Meta-Analyses Protocols (PRISMAP) Statement Guidelines.^[15]^

### 2.2 Inclusion criteria for study selection

Articles related to the pulmonary rehabilitation training based on WeChat App on COVID-19 patients will be included. All articles included are randomized controlled trial (RCT).

#### 2.2.1 Types of participants

Patients with confirmed diagnosis of COVID-19.

#### 2.2.2 Types of interventions

Patients in the control group were given routine treatment, while patients in the experimental group accepted the pulmonary rehabilitation training based on WeChat App on the basis of routine treatment.

#### 2.2.3 Types of outcome measures

1. Anxiety, Self-Rating Anxiety Scale or Hamilton Anxiety Scale;
2. Depression, Self-rating depression scale or Hamilton Depression Scale;
3. Life quality, related quality of life scales;
4. Lung function, forced vital capacity (FVC), and forced expiratory volume in one second (FEV1) and FEV1/FVC.

### 2.3 Data sources and search strategy

We conducted a systematic search of databases (PubMed, medRxiv, Web of Science, Scopus, Chinese Science Citation Database, China National Knowledge Infrastructure, Chinese Biomedical Literature Database, Chinese Scientific Journal Database, and Wan-fang databases) up to May 2021, using key terms. Besides, reference lists of relevant studies were identified. These search terms are summarized in Table 1.

**Table 1:** Search strategy for PubMed.

Number	Search terms
#1	Pulmonary rehabilitation [Title/Abstract]
#2	Lung rehabilitation training[Title/Abstract]
#3	Respiratory rehabilitation training[Title/Abstract]
#4	OR/1–3
#5	WeChat[Title/Abstract]
#6	Weixin[Title/Abstract]
#7	OR/5–6
#8	Corona Virus [Title/Abstract]
#9	Corona Virus Disease 2019 [Title/Abstract]
#10	COVID-19 [Title/Abstract]
#11	Novel coronavirus[Title/Abstract]
#12	Novel coronavirus pneumonia[Title/Abstract]
#13	or/8–12
#14	#4 AND #7 AND #13

### 2.4 Data collection and analysis

#### 2.4.1 Selection of studies

**Figure 1:**
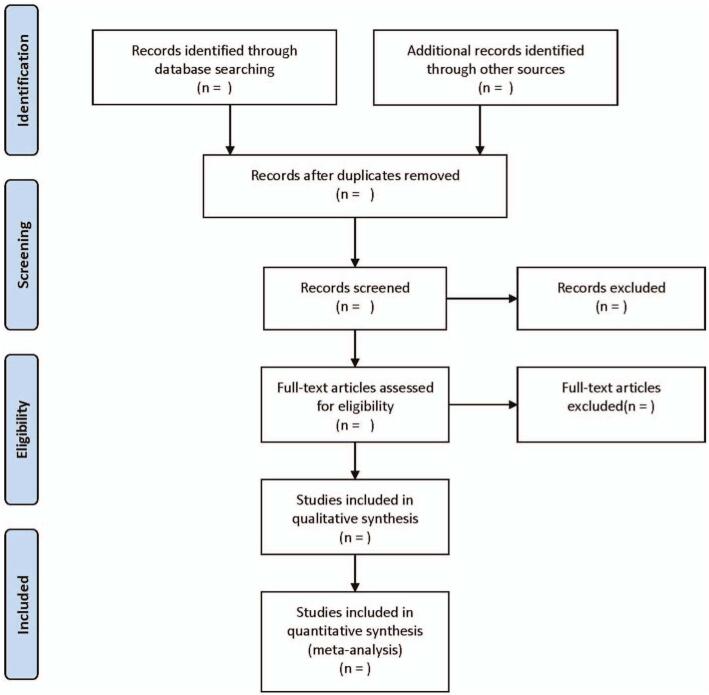
Flow diagram of study selection process.

The database search results will be imported into Endnote to delete duplicates, and then titles and abstracts will be screened to exclude references that are irrelevant to this study. Fully-text articles requiring further study will be evaluated using inclusion criteria. Two reviewers will screen the references independently and a third independent reviewer will be involved to resolve the differences in the screening process. The process of the selection is displayed in Figure 1.

#### 2.4.2 Data extraction and management

Data extraction was conducted by 2 reviewers using a standardized data extraction form. The following data were extracted: first author, year of publication, number of patients, country, age, type, content and duration of life intervention, outcomes, result measurement data, intervention time and details, etc.

#### 2.4.3 Assessment of risk of bias in included studies

The quality of randomized controlled trials included was evaluated via Cochrane Handbook which is composed of 6 items: sequence generation, allocation concealment, blinding of participants, personnel and outcome assessors, incomplete outcome data, no selective outcome reporting and other sources of bias.^[16]^ Each item will be evaluated as “high,” “low,” or “unclear”.

#### 2.4.4 Measures of treatment effects

Standardized mean difference (SMD) is applied to measure the efficacy of 95% CI.

#### 2.4.5 Management of missing data

If there are insufficient or missing data in the literature, the authors will be contacted via email. If the data are still not available, only the current available data will be analyzed and the potential impacts will be discussed.

#### 2.4.6 Assessment of heterogeneity

The heterogeneity included in the results of the study was analyzed by carrying out the χ^2^ test (the test level was α=0.1) and *I*^2^ to quantitatively determine the size of the heterogeneity. When *P* < 0.1 and/or *I*^2^ > 50%, the random effect model will be adopted for the combined analysis. Otherwise, the fixed effect model will be applied for the combined analysis.

#### 2.4.7 Assessment of reporting biases

Publication bias was detected by funnel plots if enough articles (>10) were adopted for an outcome.^[17]^

#### 2.4.8 Data synthesis

We will apply RevMan 5.3 (Copenhagen, Denmark) for data analysis and quantitative data synthesis. If there are no findings of statistical heterogeneity, the fixed-effect model will be adopted for data synthesis. If there is significant statistical heterogeneity, the random effect model will be used.

#### 2.4.9 Subgroup analysis

A subgroup analysis will be conducted on the basis of intervention time (<8 weeks or ≥8 weeks).

#### 2.4.10 Sensitivity analysis

We performed sensitivity analyses by re-estimating the pooled effects with fixed-effect or random-effect model.

#### 2.4.11 Ethical review and informed consent of patients

The content of this article does not involve moral approval or ethical review and will be presented in print or at relevant conferences.

## 3 Discussion

Studies have revealed that WeChat APP-based pulmonary rehabilitation training can effectively promote lung rehabilitation, relieve adverse emotions and improve the quality of life.^[3,18,19]^ However, to our knowledge, no meta-analysis has been conducted to evaluate the impacts of WeChat APP-based pulmonary rehabilitation training on patients with COVID19. Therefore, in order to provide new evidence-based medical evidence for rehabilitation guidance in COVID19 patients, we conducted a systematic review and meta-analysis to evaluate the impacts of WeChat APP-based pulmonary rehabilitation training on lung function, adverse mood, and quality of life in COVID19 patients. The results of this meta-analysis will provide evidence-based guidance for clinical pulmonary rehabilitation.

## Author contributions

**Conceptualization:** Aihong Xu, Lili Ding.

**Data curation:** Lili Ding, Zhiyu Xu.

**Formal analysis:** Zhiyu Xu.

**Funding acquisition:** Aihong Xu.

**Investigation:** Zhiyu Xu.

**Methodology:** Haiping Li.

**Project administration:** Aihong Xu.

**Resources:** Zhongyan Zhao.

**Software:** Zhongyan Zhao.

**Supervision:** Aihong Xu.

**Validation:** Zhongyan Zhao, Haiping Li.

**Visualization:** Haiping Li.

**Writing – original draft:** Aihong Xu, Lili Ding.

**Writing – review & editing:** Aihong Xu, Lili Ding.
